# Guaroa virus, a forgotten Latin American orthobunyavirus: A narrative review

**DOI:** 10.1371/journal.pntd.0013523

**Published:** 2025-09-19

**Authors:** Carlos Ramiro Silva-Ramos, Patricia V. Aguilar

**Affiliations:** 1 Grupo de Enfermedades Infecciosas, Departamento de Microbiología, Facultad de Ciencias, Pontificia Universidad Javeriana, Bogotá, Colombia; 2 Department of Pathology, University of Texas Medical Branch, Galveston, Texas, United States of America; 3 Center for Tropical Diseases, University of Texas Medical Branch, Galveston, Texas, United States of America; UCLM: Universidad de Castilla-La Mancha, SPAIN

## Abstract

Guaroa virus (GROV) is a neglected arthropod-borne orthobunyavirus, primarily endemic to parts of Central and South America. Its epidemiological and clinical impact remains unclear due to limited research and underreporting. It has been sporadically associated with mild febrile illness in humans. GROV is thought to be transmitted by **Anopheles* spp.* mosquitoes, but its natural reservoir hosts remain unknown. Clinically, GROV infection is characterized by fever, headache, malaise, chills, and myalgia. Co-infections with other pathogens related to febrile illnesses can occur in endemic areas. Diagnosis relies on viral isolation and reverse transcription polymerase chain reaction (RT-PCR) during the acute phase and serological testing in later stages. Although not directly oncogenic, GROV has been shown in experimental murine models to enhance the tumorigenic potential of certain oncogenic viruses; however, the underlying mechanisms remain unclear, and no evidence of this effect exists in humans. Risk factors include male gender, outdoor occupations, and living near mosquito habitats. Preventive measures focus on reducing vector contact. Future research is urgently needed to clarify GROV’s ecology and importance, including the identification of natural reservoirs, the role of *Anopheles* mosquitoes as competent vectors, and its true public health burden, particularly in rural and low-resource areas where diagnostic capacity is limited and multiple febrile illnesses co-circulate.

## 1. Introduction

Guaroa virus (GROV) was first isolated in 1956, from the bloodstream of six apparently healthy people from San Carlos de Guaroa Municipality, Meta Department, Colombia [1]. GROV was isolated from four soldiers and two civilians from the region, which did not complain about any febrile illness; however, a month later, they seroconverted [1]. Since then, GROV has been recognized as one of the neglected arboviruses linked to febrile illness in a number of tropical South American countries, most likely in Central America as well [2–5].

Although originally discovered in Colombia, phylogeographic studies by Groseth and colleagues suggest that the virus may have originated in Brazil and subsequently spread to neighboring countries, including Colombia, Peru, and Bolivia [6]. This distinction clarifies that while Colombia is the site of initial detection, molecular evidence points to Brazil as a possible source of regional dissemination [6]. Recent outbreaks of emerging arboviruses such as Oropouche virus (OROV), also endemic to Central and South America, have drawn considerable public health attention due to their epidemic potential and impact on local populations [7,8]. However, GROV remains largely neglected and poorly studied despite its circulation in similar geographic areas, with limited information available focused specifically on the virus. Understanding GROV ecology, epidemiology, and clinical relevance is critical to contextualize its pathogenic role among the diverse arboviruses circulating in the region. Thus, the present review attempts to compile all the information currently available on GROV in order to highlight its potential role as an underrecognized cause of febrile illness, and to guide future research addressing critical gaps in understanding this pathogen, with particular attention to rural or resource-limited areas, where diagnostic tools are scarce and differential diagnosis with other febrile illnesses is especially challenging.

## 2. Methods

This narrative review is based on the available scientific literature concerning GROV. A systematic search for available literature was conducted on April 4th, 2025, in the PubMed MEDLINE, EMBASE, Scopus, and BVS databases using the keyword “Guaroa,” without any specific time range. Articles published in English, Portuguese, or Spanish that specifically addressed GROV were considered for inclusion in the present manuscript. Publications not focused on GROV were excluded.

The database search yielded 34, 38, 42, and 17 results from PubMed MEDLINE, EMBASE, Scopus, and BVS, respectively, giving a total of 131 records. After removing duplicates and excluding articles unrelated to GROV, 28 manuscripts were selected for detailed review. References cited within these 28 manuscripts were also screened to identify additional relevant publications. This secondary search resulted in the inclusion of nine additional manuscripts, bringing the final number of GROV-focused manuscripts included in this review to 37 articles.

## 3. Virology

### 3.1. Taxonomy and genomic structure

GROV (Order *Elliovirales*, Family *Peribunyaviridae*) is a member of the *Orthobunyavirus* genus, recently renamed as *Orthobunyavirus guaroense* according to the International Committee on Taxonomy of Viruses [9]. Orthobunyaviruses genome consists of three single-stranded negative-sense RNA segments: small (S), medium (M), and large (L).

The S segment encodes the nucleoprotein (N), which facilitates the viral genome encapsidation and replication. It also encodes the nonstructural protein NSs, which acts as a negative regulator of the viral replication by modulating the viral polymerase activity [9–11].

The M segment encodes the nonstructural protein NSm, which has been linked to the spread of other orthobunyaviruses from the midgut to other tissues in female mosquitoes [12–14]. It also encodes the structural glycoproteins Gn and Gc (previously known as G1 and G2, respectively), which are involved in viral invasion into the host cells [12,15].

**Fig 1:**
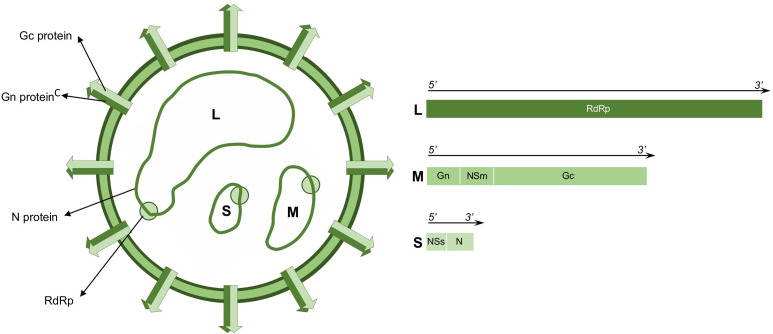
Schematic diagram of Guaroa virus viral particle and its genome.

The L segment encodes the viral RNA-dependent RNA polymerase and associated proteins with endonuclease activity, both essential for genome transcription and viral replication [15] (Fig 1).

### 3.2. Serological and phylogenetic classification

The taxonomic status of GROV has been historically controversial. Early studies based on complement fixation and immunodiffusion tests, have placed GROV within the Bunyamwera (BUN) serogroup [16,17]. However, further studies using hemagglutination inhibition (HI) and neutralization assays suggested that GROV exhibited greater antigenic similarity with the California (CAL) serogroup [1,18].

These serological discrepancies led to the hypothesis that GROV might represent a reassortant virus possessing genome segments of distinct origins from both BUN and CAL viruses [19]. However, subsequent phylogenetic analyses of the S and M segments have provided no support for a reassortant origin. Phylogenetic analyses based on S segment clusters GROV within the BUN serogroup [19], while more recent phylogenetic analysis based on the M segment positions GROV equidistantly from both BUN and CAL groups [12], rejecting the reassortment hypothesis and instead suggesting that GROV represents a unique evolutionary lineage that act as a phylogenetic bridge between the two serogroups as previously proposed [16]. Accordingly, GROV is currently classified as a distinct species within the genus *Orthobunyavirus*, not belonging to either the BUN or CAL serogroups.

Considering the antigenic relatedness of GROV to both BUN and CAL serogroup viruses, the potential for serological cross-reactivity must be carefully considered when using nonspecific assays, particularly in endemic regions where these orthobunyaviruses co-circulate. Serological cross-reactivity with orthobunyaviruses of other serogroups, such as OROV, a member of the Simbu serogroup, is less likely. Nevertheless, confirmatory and highly specific methods such as plaque reduction neutralization test (PRNT), viral isolation, or molecular approaches, should be employed whenever possible to ensure accurate results.

### 3.3. Physicochemical properties and morphology

GROV viral particles are relatively heat-labile, losing infectivity rapidly after 1 hour of exposure at 56 °C, and the virus is quite labile at 37 °C, getting completely inactivated after 24 hours of exposure at this temperature [20]. It is highly sensitive to acidic environments, with complete loss of infectivity after 2 hours of exposure at pH 2.0–4.0, whereas exposure to alkaline pH values (8.9–9.0) does not significantly affect its stability [20].

These characteristics are consistent with those of other arboviruses and suggest that GROV transmission depends strongly on direct vector–host interactions, as the virus is unlikely to remain viable outside the host under typical environmental conditions. Moreover, environmental factors such as temperature and pH, may also influence viral kinetics within mosquito vectors by modulating viral replication efficiency, similar to other arboviruses [21,22].

Ultrastructural electron microscopy revealed that GROV viral particles are ellipsoidal, approximately 70–90 nm in size, possessing a dense nucleoid and single membrane, with viral assembly occurring at the cell membrane [23].

### 3.4. Replication cycle

As with other orthobunyaviruses, GROV initiates infection by binding to host cell receptors through their surface glycoproteins, and entering through a clathrin-mediated endocytosis mechanism. Fusion between the viral Gc glycoprotein and the endosomal membrane allows the release of the nucleocapsid into the cytoplasm [24].

The 5′- and 3′- terminal regions of the viral RNA serve as promoters for the synthesis of mRNA and antigenome. Since viral mRNAs are shorter than the viral RNA and not polyadenylated, for the transcription process, a 5′-methylated cap is obtained from the host’s mRNA through cap-snatching using the viral endonuclease proteins [24,25].

Viral proteins encoded by the S and L segments are sufficient for viral replication, while specific sequences at the L segment 3′ termini are essential for transcription efficiency [26]. Translation occurs in free and membrane-bound ribosomes, after which Gn and Gc proteins are cleaved from a major polyprotein and then transported to the Golgi complex, where viral assembly and genome packaging take place, since nucleoproteins are also located near this region. This process is guided by signals found in non-conserved sequences within the terminal untranslated regions. Mature virions emerge from the Golgi cisternae and are transported to the cell membrane for release via the secretory pathway [24].

## 4. Endemic area

### 4.1. Geographic origin and spread

**Fig 2:**
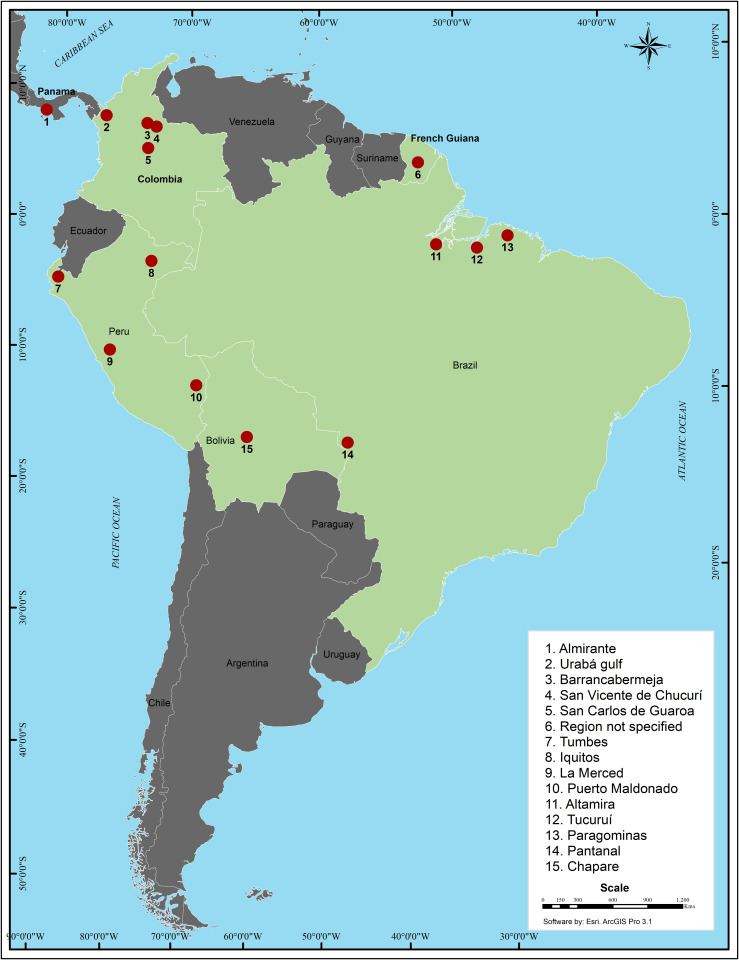
Map with the regions where Guaroa virus (GROV) is endemic. Countries in green are the ones where the circulation of GROV has been detected. Red dots with numbers are the specific regions within each country where data on the circulation of GROV has been obtained. Each number refers to the specific region detailed within the dashed box. Map has been created in ArcGIS Pro 3.1 under an academic license.

GROV is actively circulating throughout Bolivia, Brazil, French Guiana, Colombia, Panamá, and Perú (Fig 2). While GROV was first isolated in Colombia, spatiotemporal analysis suggested that the common ancestor of GROV and Wyeomyia virus, a member of the BUN serogroup, was introduced to South America approximately 250 years ago from Africa, most likely in an area 250 km from Manaus city in Amazonas State, Brazil, along the Amazon River [5].

Several important events, including independence wars and insurrections in this area, occurred during European colonization, making it a historical hotspot. These events might have contributed to the spread of several viruses, including GROV [27–29]. The study suggests that GROV initially spread north to northern Brazil and Colombia, before moving south to Peru and Bolivia, which are regarded as recently infected regions [6].

It is likely that GROV cases are going undetected in the region, not only due to limitations in existing surveillance systems, but mainly because active surveillance for GROV is practically nonexistent. Public health efforts tend to focus on higher-priority arboviral infections, often leaving emerging or neglected viruses like GROV under the radar. In this context, targeted surveillance efforts led by research groups and academic institutions may represent the most viable approach to improve detection and generate evidence that could eventually help define the true burden and clinical relevance of GROV in endemic regions.

### 4.2. Colombia

In San Carlos de Guaroa Municipality, a seroprevalence rate of 43.6% (48/110) was documented among the local population, including 41 military personnel and 69 civilians, who were sampled in the context of an investigation conducted following reports of febrile illness in the area [1]. GROV was later isolated from febrile patients in other regions of Colombia. Additional studies performed in other regions of Colombia confirmed active GROV circulation. In the Santander Department, a seroprevalence rate of 64% (34/53) and 47% (73/154) was observed among local residents from San Vicente de Chucurí and Barrancabermeja municipalities, while nearly half of the population in the Urabá Gulf Region had serological evidence of prior exposure to GROV [2].

### 4.3. Brazil

In Brazil, GROV is considered the second most widespread orthobunyavirus in the Amazon Region, after OROV [3]. GROV was first isolated in Brazil from a human case in 1986 [30], and a seroprevalence rate of 18% was detected in several localities from the Amazon basin [31]. GROV was isolated from humans in Altamira Municipality, Pará State, but no neutralizing antibodies were found in the same area among the local population [32].

Co-infections between GROV and *Plasmodium vivax* have also been documented in Paragominas and Tucuruí municipalities [33]. In Tucuruí Municipality, GROV was isolated from both humans and mosquitoes, *Anopheles nuneztovari* and *A. triannulatus* species, identifying also cases of seroconversion [32]. Additional studies investigating the circulation of GROV in animals have also been conducted in the same region, identifying previous exposure among *Alouatta belzebul* (Red-handed howler) monkeys captured during flood periods [32]; and in the Pantanal Region, Matto Grosso Do Sul State, anti-GROV antibodies were detected among 0.4% (1/232) of sheep sampled [34].

Numerous strains have been isolated from a variety of hosts in various parts of the nation, the majority of which were febrile patients, indicating that it may be involved in the regional etiologies of tropical disorders in Brazil [3,35].

### 4.4. Peru

According to studies conducted in Peru, GROV has been detected in several regions, mainly in the northern Peruvian Amazon Region, where it is considered one of several neglected emerging arboviruses [36,37]; however, it is unclear whether this reflects active geographic spread or simply increased detection due to focused surveillance efforts in the area.

In 1972, serological evidence against GROV was found in 70% (70/100) of the residents from the half of Peru lying east of the eastern foothills of the Andes in the Peruvian Amazon Region using HI assays [38]. In 1995, GROV was first isolated from a patient with acute undifferentiated febrile illness (AUFI). Fifteen confirmed and thirty presumptive cases of GROV were reported between 2000 and 2009, including three from Iquitos, Maynas province, Loreto Department, where a 2010 investigation discovered a 13% (144/1124) seroprevalence rate among the local population [4].

Additionally, samples from febrile patients from Madre de Dios Department, Junin, and Tumbes revealed the presence of GROV [4]. More recently, a 2020 investigation found that GROV was the cause of AUFI in 11.6% (14/121) of febrile patients from Iquitos over a 3-month period. All of them were tested for different arboviruses, but seroconverted only to GROV, and samples from these patients yielded eleven GROV isolates [5].

### 4.5. Bolivia

Currently, the only available data in Bolivia is the confirmation of two cases of febrile illness due to GROV in a male agricultural worker and a female student, both of whom were reported in the Chapare Region, Cochabamba Department [4].

### 4.6. Other countries

Human infections or exposure to GROV have not been reported in other American countries. However, in the Almirante district, Boca del Toro province, Panama, GROV was isolated from a mixture of several *Anopheles* species captured in the region [39,40] and from *A. peryassui* species sampled from French Guiana [41,42].

The negative results to identify prior exposure to GROV outside of the endemic region in South America, such as in Sri Lanka, where antibodies against GROV have not been detected, corroborate the geographic restriction of GROV to a few South American countries [43].

## 5. Vectors and natural reservoirs

As an arthropod-borne virus, GROV has been detected in several mosquito species. Unlike most arboviruses, which are typically transmitted by *Aedes* or *Culex* mosquitoes, GROV is uniquely associated with *Anopheles* mosquitoes, which appear to be the primary vectors contributing to its transmission. According to a 1989 study carried out in Brazil, GROV was isolated from both *A. triannulatus* and *A. nuneztovari* mosquitoes, as well as from a human during the same flooding period, providing evidence of simultaneous circulation in vectors and humans [32]. Both *A. triannulatus* and *A. nuneztovari* inhabit forested or rural areas and exhibit anthropophilic behavior, biting primarily at night both indoors and outdoors, with the latter also recognized as a competent malaria vector in South America. In Panama, GROV has also been detected in *Anopheles* mosquitoes, although the specific species were not identified [39,40], and in French Guiana, the virus was found in *A. peryassui* mosquitoes, a species of sylvatic habits frequently associated with forest margins [39,41]. In Colombia, despite the extensive entomological surveillance and the simultaneous collection of multiple mosquito genera in the Pacific lowlands, GROV has only been successfully identified from *A. (Kerteszia) neivai*, a species closely associated with humid forest areas that can have an anthropophilic behavior [44]. These findings highlight the relevance of *Anopheles* spp. mosquitoes in the ecology of GROV.

The natural reservoir host of GROV remains unknown, as no definitive evidence or field data are currently available to identify vertebrate species that maintain the virus in nature. However, serological evidence of exposure to related orthobunyaviruses has been documented in several South American avian species, suggesting a potential role for birds in GROV ecology [45–47]. Colombia is a key strategic location along major migratory flyways for several bird species, raising the possibility that migratory birds could have introduced the virus into the region, where it might have disseminated to local bird species, which may have spread and shed GROV to other South American countries such as Peru and Bolivia [6]. Nonetheless, more ecological and virological studies are required to validate the role of avian fauna in the natural transmission cycle of GROV.

Experimental infection studies have shown that various vertebrate species can become infected with GROV under laboratory conditions. In mice of all ages, susceptibility to GROV infection and development of viremia have been successfully demonstrated [1,48]. In nonhuman primates, including *Macaca mulatta* and *Aotus trivirgatus*, GROV infection induced the production of neutralizing antibodies after a short period of viremia, without developing disease-related symptoms [1]. Similarly, donkeys experimentally infected with GROV did not exhibit clinical signs or detectable viremia, but they did seroconvert and develop neutralizing antibodies [49]. Collectively, these findings suggest that a range of vertebrate species may be susceptible to GROV infection and could potentially participate in its eco-epidemiology; however, their specific roles as amplifying or reservoir hosts remain to be elucidated.

## 6. Pathogenesis

The pathogenesis of GROV remains incompletely understood. It is known that GROV is a cytopathic virus capable of inducing degenerative effects in various cell types, including embryonic lung strains, HeLa, HEp-2, Chang-conjunctiva cells, embryonic kidney cells, and most notably, bovine lymph node cells [20].

*In vitro* studies demonstrate that GROV infection leads to a progressive increase in viral infectivity over a 4-day period, with cytopathic effects such as cellular degeneration and lysis appearing as early as 72 hours postinfection [23]. GROV replicates very actively within the infected cells [23]. Viral antigens can be occasionally detected as early as day 1, becoming more abundant over time and typically accumulating near the cell membrane as sharply defined cytoplasmic particles [23]. Neutralizing antibodies typically appear between days 9 and 14 postinfection and reach peak levels by day 21 lasting for at least 16 weeks [38,49].

Interestingly, although GROV itself is considered nononcogenic, experimental studies in mice have suggested that GROV may enhance the tumorigenic potential of murine sarcoma virus, through a mechanism not fully elucidated, but appears to involve genetic interactions between GROV and the oncogenic virus, potentially leading to increased oncogenesis [50–52]. Notably, this effect has not been widely observed with other non-oncogenic viruses [53]. However, it is important to emphasize that these findings are based solely on murine models, and no evidence to date supports a similar phenomenon in humans. Thus, while intriguing, these results should be interpreted with caution and warrant further investigation, particularly in endemic areas where GROV is actively circulating and co-infections with other pathogens may occur.

## 7. Clinical manifestations

Only one of the six individuals from whom GROV was initially isolated from San Carlos de Guaroa in Colombia, showed symptoms of a mild febrile illness during the month-long follow-up period [1]. Additional studies have demonstrated that GROV viremia occurs at low levels during the first 3–4 days postinfection [48].

The diagnosis of GROV infection is made more difficult by the fact that its clinical symptoms are generally mild, nonspecific, and indistinguishable from those of other febrile illnesses, particularly in tropical regions where a number of AUFI-associated pathogens are also common [4]. The most frequently clinical manifestations include fever, which usually lasts between 4 and 5 days, and nonspecific symptoms such as headache, chills, malaise, myalgia, and arthralgia [4,5]. Table 1 summarizes the information on clinical manifestations that occurred during GROV infection. However, given that only 27 GROV infections with clinical data have been reported in the literature to date, 21 mono-infections and 6 co-infections [4,5], caution must be exercised when interpreting these findings, as the small sample size limits the ability to draw broad conclusions about the full spectrum of clinical manifestations, severity, or coinfection dynamics.

**Table 1:** Clinical features of Guaroa virus (GROV) mono and co-infection (adapted from Aguilar and colleagues [4] and Siles and colleagues [54]).

	GROV mono-infection (*n* = 21) (Aguilar and colleagues [4]; Siles and colleagues [54])	GROV and *Plasmodium vivax* co-infection (*n* = 6) (Siles and colleagues [54])
**Fever**	21/21 (100%)	6/6 (100%)
**Headache**	20/21 (95.2%)	6/6 (100%)
**Malaise**	20/21 (95.2%)	6/6 (100%)
**Chills**	19/21 (90.5%)	6/6 (100%)
**Myalgia**	18/21 (85.7%)	5/6 (83.3%)
**Arthralgia**	17/21 (81%)	6/6 (100%)
**Bone pain**	16/21 (76.2%)	6/6 (100%)
**Retroorbital pain**	14/21 (66.7%)	5/6 (83.3%)
**Nausea**	11/21 (52.4%)	5/6 (83.3%)
**Conjuctival injection** ^*^	10/21/47.6%)	2/6 (33.3%)
**Dysgeusia**	8/21 (38.1%)	4/6 (66.7%)
**Anorexia**	7/21 (33.3%)	6/6 (100%)
**Abdominal pain**	7/21 (33.3%)	3/6 (50%)
**Asthenia**	6/21 (28.6%)	0/6 (0%)
**Dizziness**	5/21 (23.8%)	4/6 (66.7%)
**Rhinorrhea**	5/21 (23.8%)	0/6 (0%)
**Rash**	4/21 (19%)	1/6 (16.7%)
**Cough**	4/21 (19%)	0/6 (0%)
**Vomiting**	4/21 (19%)	0/6 (0%)
**Diarrhea**	3/21 (14.3%)	1/6 (16.7%)
**Sore throat**	2/21 (9.5%)	0/6 (0%)
**Arthritis**	1/21 (4.8%)	0/6 (0%)
**Ear pain**	1/21 (4.8%)	0/6 (0%)
**Expectoration**	1/21 (4.8%)	0/6 (0%)
**Petechiae**	1/21 (4.8%)	0/6 (0%)
**Weight loss**	1/21 (4.8%)	0/6 (0%)
**Gingivorrhagia**	0/21 (0%)	1/6 (16.7%)

* Conjunctivitis report from Siles and colleagues (2020) was unified into conjunctival injection.

Since several arboviruses and other pathogens related to AUFI are also circulating in regions where GROV is endemic, co-infections are not rare. Co-infections between GROV and malaria parasites *P. falciparum* and *P. vivax* have already been reported [5,33]. Although the duration of the fever and other nonspecific symptoms is not different between GROV mono-infections and co-infections, in some cases, clinical manifestations such as headache can persist for a longer period of time, and clinical manifestations such as conjunctival injection, dysgeusia, cough, and sore throat are apparently more common in GROV mono-infections [5]. Considering that the disease caused by GROV infection is usually mild, most patients do not require hospitalization and recover without any long-term sequelae; however, in co-infections, GROV could be a cause of long-term symptomatology such as persistent fever [5].

## 8. Diagnosis

GROV isolation remains as the most definitive method for confirming active infection, as it provides direct evidence of the virus; however, successful isolation is not always achievable in all cases [54]; therefore, molecular techniques that detect the viral RNA and serological methods demonstrating seroconversion are commonly employed as complementary alternatives for diagnosis. GROV can be cultured in various cell lines, including HEp-2 and Vero cells, where it induces noticeable cytopathic effects typically within 3 days postinfection [4,23]. Early indicators of viral replication include increased cytoplasmic RNA staining and the appearance of dense globular aggregates within degenerating cells as early as 24 hours after inoculation, as well as the detection of viral antigens by immunostaining, often concentrated near the cell membrane [23]. Electron microscopy can further confirm infection by revealing the presence of ellipsoidal virions assembling at the host cell membrane [23]. Patient serum samples are usually used for viral isolation during the viremic phase; these are diluted and inoculated into established cell cultures, which are then monitored for the appearance of cytopathic effects, serving as indicators of active viral replication [4].

Molecular methods offer a valuable complementary strategy to confirm GROV diagnosis, mainly during the early stages of infection when viral RNA is still detectable on serum samples due to viremia, and can be performed before the antibody response is measurable [5]. RT-PCR has proven effective in directly identifying GROV RNA in clinical samples. RT-PCR protocols using different sets of primers targeting conserved regions of the S, M, and L genome segments have demonstrated the capacity to detect multiple orthobunyaviruses, including GROV [4,55,56]. Molecular testing serves as a powerful diagnostic tool, particularly in areas with co-circulating arboviruses, where serological cross-reactivity can occur and may limit the specificity of the methods employed; and when used together with serology and viral isolation, molecular methods significantly enhance the accuracy and chances of GROV detection [5].

GROV diagnosis also relies on serological methods, which aim to detect viral-specific antibodies in patient serum samples. In clinical and epidemiological settings, seroconversion demonstrated by a 4-fold or greater increase in IgM titers between acute and convalescent phases by PRNT, remains as the gold standard and key method for diagnosis, particularly among patients presenting with AUFI when viral isolation is not possible [54]. HI assays can be employed for initial antibody screening; however, these do not necessarily reflect functional immunity; in contrast, neutralization assays, such as the PRNT, offer greater specificity and provide a more accurate assessment of protective antibody responses, being a highly helpful tool for specific GROV diagnosis [4,38]. A combination of viral isolation along with serological assays, PCRs, and sequencing can aid in detecting GROV, and additionally, IgM capture ELISAs targeting GROV followed by PRNT can also confirm recent infection [54].

Unfortunately, despite the availability of virological, molecular, and serological methods for GROV diagnosis, their clinical application in endemic regions is often unfeasible. Most techniques require specialized equipment, highly trained personnel, and well-equipped laboratories, which are rarely accessible in rural or low-resource areas. Moreover, GROV infection presents with nonspecific symptoms that overlap with other causes of AUFI (e.g., dengue, leptospirosis, OROV). Therefore, although differential diagnosis is essential, it is frequently limited by the lack of appropriate diagnostic tools, the syndromic similarity with other infections, and the limited awareness among healthcare professionals about neglected pathogens like GROV. Additionally, the scarcity of clinical studies hampers a detailed characterization of the disease, including laboratory parameters that could support its initial clinical suspicion.

## 9. Treatment

Although the disease caused by GROV is considered mild, the limited number of studies conducted to date prevents a full understanding of its clinical significance. It remains unknown whether GROV could be implicated in future outbreaks or pose a greater risk to specific populations, such as immunocompromised individuals, who may require targeted therapeutic approaches. Further research is essential to address these uncertainties.

Currently, there is no antiviral treatment officially validated or approved for clinical use against GROV. Several compounds with known antiviral activity have been tested *in vitro* for their efficacy against GROV and other orthobunyaviruses. Notably, mycophenolic acid and ribavirin, two agents with broad-spectrum antiviral properties, have failed to effectively inhibit GROV replication, indicating limited potential for their use in GROV treatment [57,58].

IFN-α, widely used in the clinical management of chronic hepatitis B and C [59], has shown promising results to suppress replication of a range of viruses, including Dengue [60] and Ebola [61] viruses. Experimental studies on GROV showed that IFN-α can significantly reduce its replication both *in vitro* and *in vivo* [58]. However, despite these encouraging preliminary findings, its clinical application remains questionable since IFN-α is associated with considerable side effects [62], and given that GROV typically causes mild illness, the use of this potentially toxic agent may not be justified.

## 10. Risk factors and prevention measures

In regions where GROV is actively circulating, certain demographic and environmental factors have been associated with a higher risk of exposure and infection. Epidemiological studies suggest that the male population with a mean age of 28 years old has an increased risk, likely due to occupational and behavioral patterns which increase their interaction with vector habitats [4]. As expected for many infections endemic to a given area, the risk of GROV exposure appears to increase with age, likely reflecting cumulative contact with the pathogen over time, since serological surveys have consistently shown lower seropositivity rates in children compared to adults in endemic areas [4].

Occupational exposure is a significant determinant of risk for GROV infection, particularly due to the virus’s sylvatic transmission cycle. Individuals whose work involves to stay for a prolonged time in forested or surrounding environments, such as woodcutters, fishermen, agriculturists, and oil-field workers, are more likely to be exposed to mosquitoes [4]. Vector activity, particularly of anopheline species which are suspected to be involved in GROV transmission, typically begins at dusk and continues through the night, placing people who work or travel during twilight or overnight in endemic zones at higher risk [4]. Environmental and socioeconomic conditions also play an important role for GROV exposure. Individuals living in wooden houses or near rivers face a higher risk compared to those in brick homes or urbanized areas with better infrastructure conditions [4]. While such factors are common to other arboviruses, they are particularly relevant to GROV, given its current restriction to rural and forest-adjacent regions.

Since GROV remains a neglected arbovirus and it has not been implicated in large-scale outbreaks, no specific prevention or control strategies have been established exclusively for GROV [3]. Reducing human exposure to mosquito vectors, is the most effective way to avoid infection [63]. Vector control is a way of reducing human exposure to mosquitoes. Although little is known about the specific mosquito species responsible for GROV transmission, general vector control programs may help limit its spread.

## 11. Discussion and future perspectives

The true and real global burden and importance of GROV remains unclear to date. Underreporting is quite common since GROV asymptomatic or mild infections occur frequently, especially in places with poor access to medical care and diagnostic tools. This is made more difficult by the striking similarities in clinical presentation with other endemic tropical febrile illnesses. Therefore, there is a need for further studies to evaluate the true impact of GROV on populations living in endemic areas. While *Anopheles* mosquitoes have been suggested as potential vectors in some areas, confirming their role in GROV transmission requires more in-depth vector competence studies. All of these studies might provide insights into GROV and its public health relevance.

Despite its discovery several decades ago, GROV remains as one of the most understudied arboviruses in the Americas. The limited number of publications, coupled with diagnostic challenges and underreporting, has made it difficult to clearly determine its true relevance. In many endemic areas, infections caused by GROV are likely misclassified within the broad spectrum of AUFI etiologies, due to the clinical overlapping and the lack of specific diagnostic tools. These knowledge gaps significantly limit the capacity to address the burden of disease attributable to GROV and to anticipate its potential for emergence.

Although GROV has not caused outbreaks of similar magnitude, the case of OROV, an orthobunyavirus initially overlooked and currently responsible for notable outbreaks in South America, may serve as an early warning [7,8]. The emergence of OROV as a public health concern underscores the need to anticipate similar risks posed by lesser-known arboviruses such as GROV, which, although apparently less pathogenic, its true burden remains unclear due to the limited studies focused on this arbovirus. Consequently, there is an urgent need for well-designed studies to better characterize the epidemiology and clinical impact of GROV in endemic populations.

Future research should prioritize longitudinal seroepidemiological studies to monitor infection dynamics, seroconversion rates, and potential seasonal patterns over extended periods. Such studies will provide valuable data on incidence, immunity, and risk factors associated with GROV infection. Equally important are comprehensive vector competence studies not only among *Anopheles* mosquitoes, but also in other mosquito species that are also important for public health. These experiments should assess the capacity of different mosquito species to acquire, maintain, and transmit GROV between different hosts.

Additionally, the development, optimization, and validation of sensitive and specific molecular diagnostic assays that can be easily employed in low-resource regions are critical. Rapid and reliable diagnostic methods will improve case detection, outbreak response, and surveillance systems. Integration of these approaches with the existing surveillance networks on AUFI will facilitate differentiation of GROV infections from other pathogens.

Together, these proposed research directions will fill critical knowledge gaps, providing a clearer and strong knowledge of GROV ecology, transmission dynamics, clinical burden, and public health significance. Such efforts will be essential for informing targeted intervention strategies in endemic regions. Importantly, incorporating GROV testing into AUFI surveillance studies, mainly among samples that tested negative for more common and prevalent arboviruses, could improve the understanding of GROV’s true burden in endemic regions.

## 12. Limitations of the review

Despite efforts to compile and synthesize the most comprehensive information available on GROV, the present review remains limited in its ability to formulate broad or definitive conclusions due to the scarce, fragmentary, and limited scientific data specifically focused on this virus. This limitation reflects the broader neglected status of GROV within the field of arbovirology. Knowledge gaps have been acknowledged throughout the manuscript, and the available findings have been interpreted with appropriate caution. This review should be considered as an initial step toward consolidating existing evidence and underscoring the urgent need for additional studies and enhanced surveillance in regions where GROV is suspected to circulate.

**Key learning points**

- Guaroa virus (GROV) is a neglected orthobunyavirus endemic to Central and South America, with an unclear epidemiological and clinical impact due to limited research and frequent underreporting.
- GROV infections often present with nonspecific febrile symptoms, making diagnosis difficult, mainly in rural or low-resource settings where multiple similar tropical diseases co-circulate.
- Current diagnostic tools include viral isolation, RT-PCR, and serological testing; however, these methods are not widely accessible in endemic areas, contributing to diagnostic gaps and underestimation of GROV’s burden.
- While not directly oncogenic, GROV has been shown in experimental models to enhance the tumorigenic potential of other viruses, although this effect has not been demonstrated in humans.
- Further research is needed to clarify the ecology of GROV, identify natural reservoir hosts and competent vectors, and determine its true public health impact in endemic regions where it is present.
